# Promoter Hypomethylation Is Responsible for Upregulated Expression of HAI-1 in Hepatocellular Carcinoma

**DOI:** 10.1155/2019/9175215

**Published:** 2019-08-28

**Authors:** Xiaoxiao Du, Lingyan Wu, Muhammad Saif Ur Rahman, Xiaodong Teng, Lisong Teng, Jingjia Ye, Jiang Cao

**Affiliations:** ^1^Clinical Research Center, The Second Affiliated Hospital, School of Medicine, Zhejiang University, Hangzhou, Zhejiang 310009, China; ^2^Department of Surgical Oncology, The First Affiliated Hospital, School of Medicine, Zhejiang University, Hangzhou, Zhejiang 310003, China; ^3^Department of Pathology, The First Affiliated Hospital, School of Medicine, Zhejiang University, Hangzhou, Zhejiang 310003, China; ^4^Chu Kochen Honors College, Zhejiang University, Hangzhou, Zhejiang 31005, China

## Abstract

An upregulated expression of hepatocyte growth factor activator inhibitor type 1 (HAI-1) in hepatocellular carcinomas (HCC) associates with poor prognosis, but the underlying mechanism for expression regulation has not been elucidated. HAI-1 was expressed in HCC cell line Hep3B cells at a high level but absent or has a low level in other HCC cell lines HepG2 and SMMC7721 and immortal normal liver cell line L02 at transcriptional and translational levels, respectively. A dual-luciferase reporter assay showed that transcriptional activity of HAI-1 in the promoter region (-452 bp to -280 bp from the mRNA start site) was strongly enhanced in Hep3B and SMMC7721. Bisulfite genomic sequencing results of the HAI-1 promoter region showed an inverse correlation between levels of promoter methylation and expression in HCC cells. The expression level of HAI-1 in SMMC7721, HepG2, and L02 cells was elevated after 5-Aza-2′-deoxycytidine treatment. Hypomethylation of the HAI-1 promoter region contributed to the elevated HAI-1 expression in HCC tissues. In addition, the hypomethylation of the HAI-1 promoter region correlated with poor differentiation status of HCC tissues. Our findings indicate that promoter hypomethylation is an important mechanism for aberrant HAI-1 expression regulation in HCC.

## 1. Introduction

Liver cancer is the second leading cause of death worldwide and the third in China [1, 2], with an increased death rate at a faster pace than any other types of cancer in recent years [3]. Hepatocellular carcinoma (HCC) represents the major histological subtype of primary liver cancer. Despite recent advances in diagnosis and survival in liver cancer, the prognosis of HCC patients remains unsatisfactory due to postsurgical recurrence, distant metastasis, and poor response of patients to conventional chemotherapy [4, 5]. The neoplastic revolution of HCC is thought of as a complex multistep process involving both genetic and epigenetic alterations eventually culminating malignant liver cancer disease [6]. Thuswise, to illustrate the functional characterization of an epigenetic mechanism in hepatocarcinogenesis which has not been fully elucidated could inform biomarker discovery and therapy for the clinical management of this malignancy.

Hepatocyte growth factor activator inhibitor type 1 (HAI-1, official name SPINT1 for serine protease inhibitor type 1) is a membrane-bound Kunitz-type serine protease inhibitor, encoded by the SPINT1 gene. HAI-1 was initially identified as the inhibitor of hepatocyte growth factor activator (HGFA) [7], subsequently found to inhibit several type II transmembrane serine proteases (TTSPs), including matriptase, prostasin, hepsin, transmembrane protease serine 13 (TMPRSS13), human airway trypsin-like protease (HAT), KLK-4, KLK-5, and human airway trypsin-like protease 5 (HATL5, HAT-like 5) [7–12]. HAI-1 has been shown to be essential to the integrity of the basement membrane during placental development [13, 14]. Loss of HAI-1 is associated with placental differentiation, prenatal lethality [15–17], decreased intestine barrier function [18], and epidermal keratinization [19, 20] in mice. To date, several studies have revealed that the expression of HAI-1 was significantly downregulated in many carcinomas indicating possible roles in carcinogenesis [21], metastasis [22], and invasion [23]. In breast cancer, low expression or knockdown of HAI-1 enhanced migration, proliferation, and metastasis [24]. A pancreatic cancer cell-derived orthotopic xenograft model, after the loss of HAI-1, was characterized by increased invasiveness and metastasis [25]. HAI-1-deficient oral squamous cell carcinoma (OSCC) cell lines showed increased migration mediated by activation of protease-activated receptor-2 (PAR-2) via deregulation of matriptase activities, the cognate serine protease target of HAI-1 [26]. Intriguingly, Funagayama et al. [27] found that HAI-1 was not expressed in the normal liver tissues but upregulated in the HCC patients by immunohistochemical analysis and the expression level of HAI-1 was associated with poor differentiation and prognosis for HCC patients. Both the mechanism of HAI-1 expression regulation and the roles HAI-1 may play in HCC await for extensive investigations.

Epigenetic regulation, such as DNA methylation status and histone modifications, plays important roles in regulating gene expression patterns during tumor development and progression [28]. Altered DNA methylation status has been reported in a wide range of human cancers including hepatocellular carcinoma [29]. It occurs predominantly at the position 5 of cytosine (5-mC) locating at high density of so-called CpG islands [30]. Recent studies demonstrated that abnormal DNA methylation in the promoter region results in strong transcriptional repression [31, 32]. A previous genome-wide DNA methylation study also revealed that 90% of tumors acquire either genome-wide DNA hypomethylation or CpG island methylator phenotype in hepatocellular carcinoma [6]. Other studies showed that abnormal methylation patterns of some specific genes are greatly correlated with the progression and prognosis in HCC patients [33, 34]. Hence, in contrast with the general expression pattern, it would be interesting to study whether DNA methylation status is involved in the regulation of expression activity of HAI-1 in HCC.

Therefore, in the present study, we examined the expression level of HAI-1 in several HCC cell lines. Focused on DNA methylation, to the best of our knowledge, we firstly found that HAI-1 upregulation in HCC is the consequence of hypomethylation in its promoter region. Aberrant DNA methylation of the HAI-1 promoter region correlates with the differentiation status of hepatocellular carcinoma.

## 2. Materials and Methods

### 2.1. Tissue Sample Collection

A pair of 15 patients with primary hepatocellular carcinomas and paired adjacent nontumor tissues were obtained from the First Affiliated Hospital of Zhejiang University School of Medicine (Hangzhou, China) from 2012 to 2016. All samples were collected in accordance with the policies of the Ethical Review Committee of the First Affiliated Hospital of Zhejiang University School of Medicine, and written informed consent was obtained from all the patients prior to the study. The pathological analysis was carefully confirmed by unbiased experienced pathologists.

### 2.2. Cell Lines

The human HCC cell lines Hep3B and HepG2 were obtained from ATCC (Manassas, VA, USA). The normal immortal human liver cell line L02 and human HCC cell line SMMC7721 were obtained from the Shanghai Institute of Biochemistry and Cell Biology, Chinese Academy of Sciences (Shanghai, China). Hep3B, SMMC7721, and L02 were cultured in RPMI 1640 (Booster Biological Technology Co. Ltd., Wuhan, China) medium supplemented with 10% fetal bovine serum (FBS, Biological Industries, Beit HaEmek, Israel). HepG2 was grown in Dulbecco's modified Eagle's medium (DMEM, Corning, Manassas, VA, USA) with 10% FBS. All the cell lines were maintained at 37°C and 5% CO_2_ in an incubator. 0.25% trypsin and 0.02% EDTA (Gino Biopharmaceutical Technology, Hangzhou, China) were used for a trypsinization purpose.

### 2.3. Total RNA Extraction and Quantitative Real-Time PCR (RT-qPCR)

Total RNA was isolated using a TRIzol reagent (Invitrogen, CA, USA) in accordance with the manufacturer's instructions, and cDNA was synthesized from 0.5 *μ*g of total RNA using M-MLV Reverse Transcriptase (Promega, WI, USA) and oligo dT. Quantitative real-time PCR was performed in Applied Biosystems 7500 fast Real-Time PCR Systems (Applied Biosystems, CA, USA) using GoTaq® qPCR Master Mix (Promega); specific primers used for detection of HAI-1 expression were forward primer 5′-GGCTGCCTGTGAAAAATACACGAG-3′ and reverse primer 5′-GGGCGCAGTGTTCGCTGAAG-3′. Primers used for detection of GAPDH expression which was included as a control gene to normalize gene expression were forward primer 5′-CTTAGCACCCCTGGCCAAG-3′ and reverse primer 5′-GATGTTCTGGAGAGCCCCG-3′. The expression level of HAI-1 was determined by the 2^-ΔΔCT^ method [35] after normalization to the GAPDH.

### 2.4. Western Blot Analysis

Cells were harvested from culture dishes and lysed for protein collection. Protein concentration was determined using the Pierce™ BCA Protein Assay kit (Thermo Scientific, Waltham, MA, USA). Equal amounts of total protein (45 *μ*g) were separated by 10% sodium dodecyl-sulfate polyacrylamide gel electrophoresis (SDS-PAGE) and transferred to a PVDF membrane (Millipore Corporation, Billerica, MA, USA). The membrane was blocked with 5% skim milk at room temperature for 1 h and then incubated with primary antibody (goat anti-human against HAI-1 antibody, R&D Systems, Minneapolis, USA, dilution 1 : 1000; GAPDH antibody, Santa Cruz Biotechnology, Dallas, TX, USA, dilution 1 : 10000) overnight at 4°C. The membrane was further incubated with the anti-goat secondary antibody at a 1 : 5000 dilution (Beijing Zhongshan Golden Bridge Biotechnology Co. Ltd., Beijing, China) at room temperature for 2 h. Bands were visualized by the ECL kit (Millipore Corporation) and Image Lab system (Bio-Rad, Hercules, California, USA).

### 2.5. Promoter Reporter and Dual-Luciferase Assays

Four overlapping fragments, which cover a total of 4102 bp region of human HAI-1 gene upstream mRNA start site, were cloned by PCR and used to generate a series of seven fragments with different lengths based on restriction endonuclease sites. The seven fragments were subcloned into the pGL4.10-basic reporter vector (Promega, WI, USA) and cotransfected with pGL4.74 control plasmid (Promega) into HCC cell lines. The firefly and Renilla luciferase activities were measured using the dual-luciferase reporter assay system (Promega) with a model GloMax® 20/20 Luminometer (Promega). The firefly luciferase activity value was normalized to the Renilla activity value.

### 2.6. Genomic DNA Bisulfite Conversion

The genomic DNA from HCC tissues and SMMC7721, Hep3B, and L02 cell lines were isolated using the Wizard® DNA Clean-Up System (Promega, WI, USA) and was quantified using a NanoDrop 2000 spectrophotometer (Thermo Scientific, Waltham, MA, USA). 2 *μ*g of genomic DNA was then bisulfite-converted with the EpiTect Bisulfite Kit (Qiagen, Germany) according to manufacturer's protocol.

### 2.7. Bisulfite Genomic Sequencing (BGS) Analysis

The CpG islands were detected using an online bioinformatics tool, the MethPrimer database (http://www.urogene.org/methprimer2/). The above bisulfite-modified genomic DNA of SMMC7721, Hep3B, and L02 cells was amplified by PCR. The primer set was used as follows: HAI-1 (forward 5′-A9GGGGGTAATAGTTTAATGAGTTAT-3′ and reverse 5′-TTCTCCCCCTAATTTCTAATAAAACT-3′). Bisulfite sequencing PCR products were gel-extracted and subcloned into the pGEM-T Easy Vector system (Promega, WI, USA) and transformed into Escherichia coli. Twenty individual clones were sequenced.

### 2.8. 5-Aza-2′-deoxycytidine (5-Aza-dC) Treatment

HepG2, SMMC7721, and L02 cells with a low/absent expression level of HAI-1 were seeded in 6 cm dishes at the concentration of 5 × 10^5^ cells per dish. After 24 h, cells were treated with 0.5 *μ*M to 10 *μ*M of 5-Aza-dC (Selleckchem, Houston, TX, USA), a demethylation reagent, for 24 h and 72 h for RNA and protein extraction, respectively.

### 2.9. Chromatin Condensation

SMMC7721 cells (1 × 10^4^ cells per well) were plated on coverslips in a 24-well plate and treated with different concentrations of 5-Aza-dC for 72 h. Then, cells were washed with phosphate-buffered saline (PBS) and fixed with 10% formaldehyde for 30 min at 4°C. Fixed cells were washed with PBS and incubated with 0.2% Triton X-100 for 10 min at room temperature. After washing with PBS, cells were stained with Hoechst 33342 stain (1 mg/ml) (Sigma-Aldrich, Darmstadt, Germany) and incubated for 15 min at 37°C. The cells on the coverslips were then washed with PBS for three times, after which the cells were mounted on glass slides and observed under a UV filter using a fluorescent microscope (Leica DM5500, Wetzlar, Germany). The condensed nuclei were counted against the total number of nuclei in the field, and the percentage of the condensed nuclei was calculated using Image-pro plus 6.0 software.

### 2.10. Immunohistochemical Staining

Formalin-fixed paraffin-embedded specimens of tissue specimens were cut into 4 *μ*M sections and blocked with 3% bovine serum albumin (BSA, Ameresco, OH, USA) in PBS for 1 h at room temperature after antigen retrieval by incubating for 30 min at 100°C in 0.01 M sodium citrate buffer pH 6.0 and 15 min at room temperature in 0.3% H_2_O_2_ in PBS. Sections were stained with the goat anti-human against HAI-1 antibody (R&D Systems, Minneapolis, USA) at a 1 : 200 dilution overnight at 4°C. The signals were detected by a biotinylated secondary antibody (Beijing Zhongshan Golden Bridge Biotechnology Co. Ltd., Beijing, China), and the positive reaction was observed using 3,3′-diaminobenzidine (ZLI-9019, Beijing Zhongshan Golden Bridge Biotechnology Co. Ltd.). Sections were counterstained with hematoxylin. All microscopy images were acquired on a Leica DM2500 microscope using a Leica Application Suite digital camera system. At least five random fields were selected for each specimen. The staining intensity rule was followed as a previous study [27]; two experienced pathologists performed unbiased judgment according to the percentage of hepatocytes where the cellular membrane and cytoplasm of HCC were stained: staining more than 50%, strongly positive (+++), score 3; staining in 25-50%, positive (++), score 2; staining in 5-25%, poorly positive (+), score 1; and staining less than 5%, negative (-), score 0.

### 2.11. Quantitative Methylation-Specific PCR (qMSP) and MSP Assay

Tissue DNA was isolated from 8 sections of macrodissected FFPE tumor tissues and adjacent normal liver tissues of 15 HCC patients diagnosed with different differentiation statuses. After bisulfite modification, all DNA samples were analyzed with primer sets for both methylated and unmethylated DNA by using GoTaq® qPCR Master Mix (Promega, WI, USA). The relative amount of methylation was calculated as *M* = 2^ΔCT^, ΔCT = number of copies of methylated DNA‐the number of copies of unmethylated DNA. Bisulfite treatment DNA was also used for the MSP analysis and visualized on ethidium bromide-stained agarose gel.

### 2.12. Statistical Analysis

Statistical analysis of the differences between groups was performed using GraphPad Prism 7.0 using Student's independent samples *t*-test between two groups and one-way ANOVA among different groups. Association between the methylation level of HAI-1 and clinicopathological characteristics were evaluated using the Chi-squared test in SPSS 19.0 software. In all cases, experiments had been repeated at least 3 times, and *p* < 0.05 was indicated as statistically significant.

## 3. Results

### 3.1. HAI-1 Is Expressed in HCC Cell Lines

**Figure 1:**
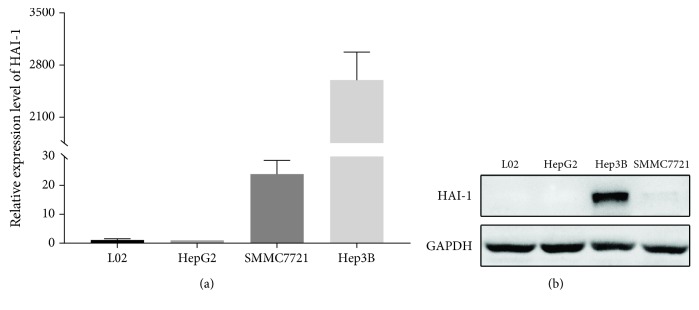
HAI-1 expression in HCC cell lines. (a) Relative HAI-1 mRNA expression levels and (b) Western blot analysis of HAI-1 protein expression in HCC and normal liver cell lines. Repeated times (*n*) = 3.

Previous studies [27, 36] revealed that HAI-1 was expressed in HCC in contrast with normal liver samples, and the existence of HAI-1 expression was associated with poor prognosis for HCC patients. To provide evidence to support further epigenetic analysis, we started with examining the mRNA expression level of HAI-1 in a panel of HCC cell lines (Hep3B, SMMC7721, and HepG2) and one normal liver cell line L02. As shown in Figure 1(a), HAI-1 was overexpressed in the more malignant HCC cell line Hep3B, decreased in less malignant HCC cell line SMMC7721, and absent in HCC cell lines HepG2 and normal liver cell line L02. Similar to the mRNA expression level, Western blotting results showed a concomitantly increased protein level of HAI-1 in the Hep3B cell line, compared with the absence or lower expression level in L02, HepG2, and SMMC7721 cell lines (Figure 1(b)).

### 3.2. Promoter Hypomethylation of HAI-1 Is Involved in HCC Cells

**Figure 2:**
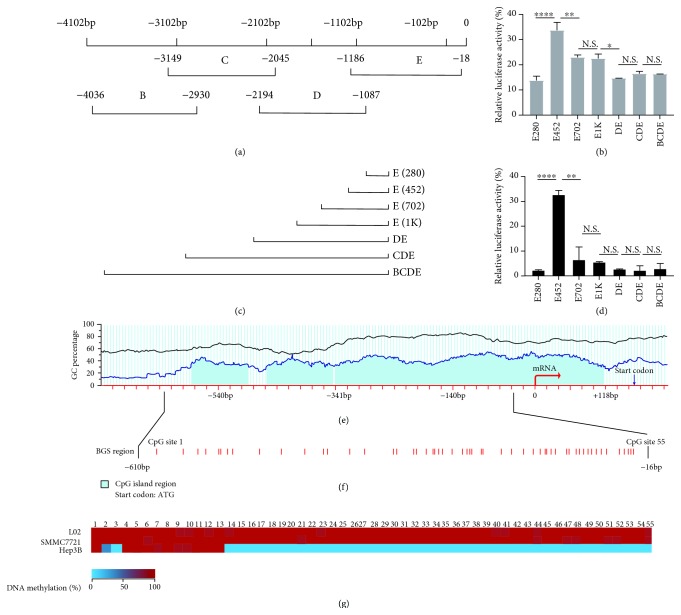
Epigenetic modification of HAI-1 in HCC cell lines. (a) The cloned HAI-1 promoter DNA fragments. The upper cartoon shows the genomic DNA, including the mRNA start site “0.” The below four lines show various fragments containing 5′ truncations of cloned HAI-1 promoter regions (but not the mRNA start site). (b) The luciferase reporter containing fragments after enzyme digestions and ligations was further transfected into Hep3B and SMMC7721 cell lines. (c, d) The relative firefly/Renilla luciferase activities of different HAI-1 promoter fragments in SMMC7721 (c) and Hep3B (d) cell lines. (N.S.: no significant difference; ^∗^*p* < 0.5, ^∗∗^*p* < 0.01, and ^∗∗∗∗^*p* < 0.0001; *n* = 3.) (e) Schematic structure of the GC content of HAI-1 predicted online (http://www.urogene.org/methprimer2/). The red arrow indicated as the mRNA start site and transcriptional direction. The blue arrow indicated as the site of start codon ATG. (f) BGS region of the HAI-1 CGIs. (g) For each cell line, 20 single clones were selected for sequencing. The average methylation frequency for each CpG site in every HCC cell line. From blue (0% methylated) to red (100% methylated).

To explore the potential biological molecular mechanism that might mediate the upregulation of HAI-1 in HCC, firstly, we cloned four HAI-1 promoter fragments upstream of mRNA start site (mRNA sequence: GenBank AB000095) from -4036 bp to mRNA start site (designated as position 0), illustrated in Figure 2(a), named B, C, D, and E clones further matched or enzyme digested into seven fragments (Figure 2(b)). Dual-luciferase report assays were performed in Hep3B and SMMC7721 after transfection to validate different promoter region activities. The results showed that the transcriptional activity of both SMMC7721 (Figure 2(c)) and Hep3B (Figure 2(d)) in -452 bp to -280 bp from mRNA start site was strongly enhanced (*p* < 0.0001) but significantly decreased among -702 bp to -452 bp (*p* < 0.0001). These results suggested a strong enhancer and a silencer in the two regions, respectively. Therefore, we focused on ~500 bp upstream promoter region of HAI-1 for further study.

Given that DNA methylation was extensively involved in the promoter region to alter specific gene activation, we used an online bioinformatics tool (http://www.urogene.org/methprimer2/) and analyzed the region around the mRNA start site (-700 to +240 bp) of the HAI-1 gene. The HAI-1 promoter region was located in a typical CpG island (CGI) spanning -341 to +118 bp (Figure 2(e)). On the basis of the HAI-1 CGI sequence, we examined the detail methylation profiles of HAI-1 CGI by BGS analysis of 55 CpG sites (Figure 2(f)). We analyzed the methylation frequency in each CpG sites (Figure 2(g)) and average methylation status over all sequences in these three cell lines (Table 1). The results revealed that the methylation level of HAI-1 was higher in L02 cell lines (96.4%) and SMMC7721 cell lines (97.6%) than in Hep3b cell lines (21.0%). Compared to the expression level of HAI-1, these results revealed a direct correlation between the methylation level of the HAI-1 promoter region and HAI-1 expression in HCC cell lines.

### 3.3. Demethylation Leads to HAI-1 Expression in HCC Cells

**Figure 3:**
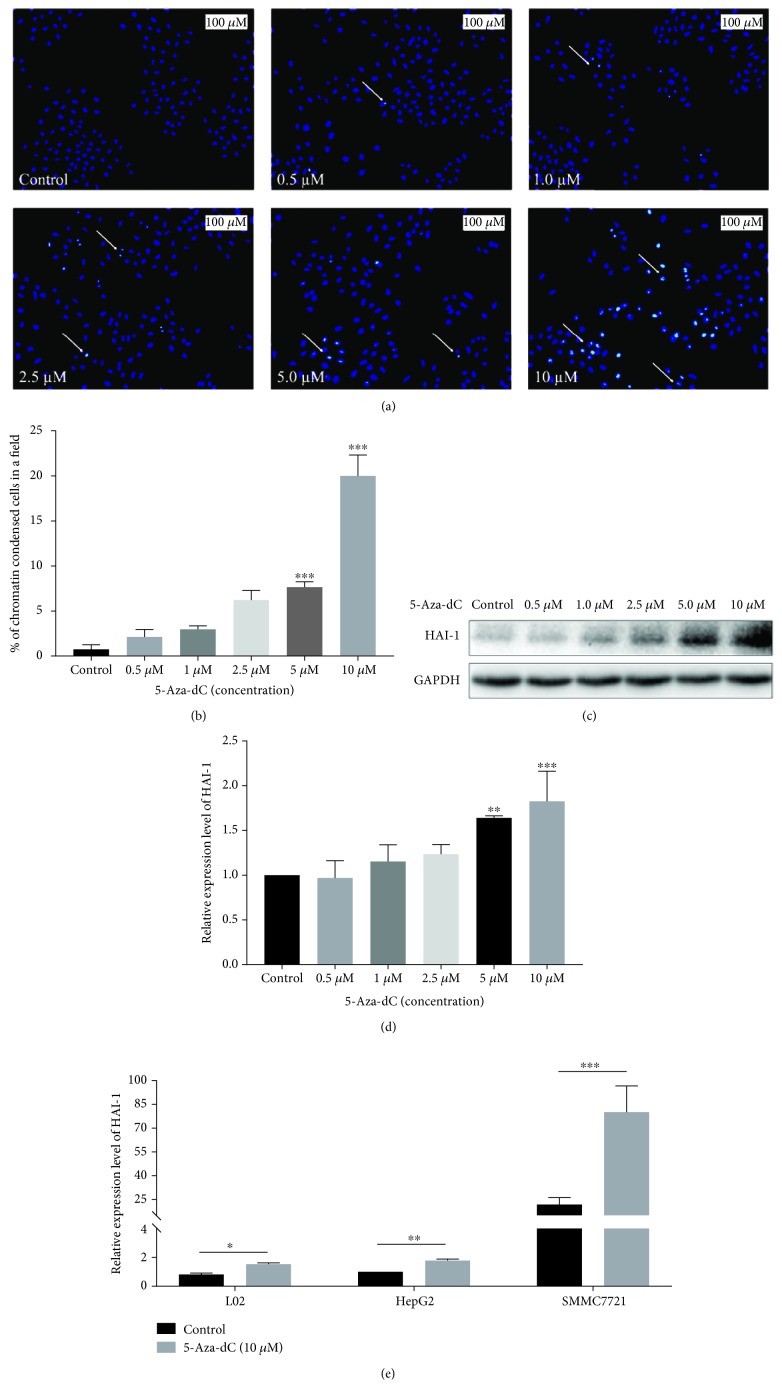
HAI-1 expression is upregulated after demethylation in SMMC7721 cell line. (a) Chromatin condensation assay: SMMC7721 HCC cell line was stained with Hoechst 33342 after 5-Aza-dC treatment (0.5 *μ*M to 10 *μ*M) for 72 h. Arrow: the representative images of stained nuclei. *n* = 3. (b) Percentage of condensed nuclei is represented graphically (mean ± SD, ^∗∗∗^*p* < 0.001, *n* = 3). Scale bar = 100 *μ*m. Protein (c) and mRNA (d) levels of HAI-1 expression in the SMMC7721 cell line were upregulated in a dose-dependent manner after 5-Aza-dC treatment for three days and one day, respectively. (^∗∗^*p* < 0.01; ^∗∗∗^*p* < 0.001, *n* = 3.) (e) Alternation of HAI-1 mRNA expression level in L02, immortal normal liver cell line, and HepG2 and SMMC7721, two HCC cell lines, with 10 *μ*M 5-Aza-dC for 24 h (mean ± SD). *n* = 3.

To investigate whether the expression level of HAI-1 would upregulate after demethylation in HCC, we firstly performed the chromatin condensation assay using Hoechst 33342 stain and observed that nuclei of 5-Aza-dC-treated, which is a DNA methyltransferase inhibitor, SMMC7721 cells showed highly stained and condensed chromatin bound to fluorescence dyes under a fluorescence microscope (Figure 3(a)). The percentage of the condensed nucleus was significantly increased in a dose-dependent manner in the presence of 5-Aza-dC (Figure 3(b), 7.64% in the 5 *μ*M group versus 0.73% in the control group, *p* < 0.0001; 20.0% in the 10 *μ*M group versus 0.73%, *p* < 0.0001). This allowed clear discrimination from untreated SMMC7721 cells with normal nuclei. We next examined HAI-1 expression changes in SMMC7721 cells after treatment with 5-Aza-dC. Western blot results (Figure 3(c)) showed that the protein expression level was increased in response to 5-Aza-dC in a dose-dependent manner, especially in the 10 *μ*M 5-Aza-dC compared with untreated cells. Consistent with the protein expression level of HAI-1, the mRNA expression level of HAI-1 was significantly upregulated after 5 *μ*M (*p* = 0.0019) and 10 *μ*M (*p* = 0.0001) 5-Aza-dC treatment, for 24 h (Figure 3(d)). Similarly, demethylation-induced upregulation of HAI-1 was found in L02 (*p* = 0.017) and HepG2 cells (*p* = 0.009) (Figure 3(e)). Taken together, these results strongly suggest that downregulation of HAI-1 in HCC is associated with its promoter hypermethylation.

### 3.4. Promoter Hypomethylation Status Is Responsible for Upregulated Expression of HAI-1 in HCC Patients and Is Associated with Poor Tumor Differentiation in HCC Patients

**Figure 4:**
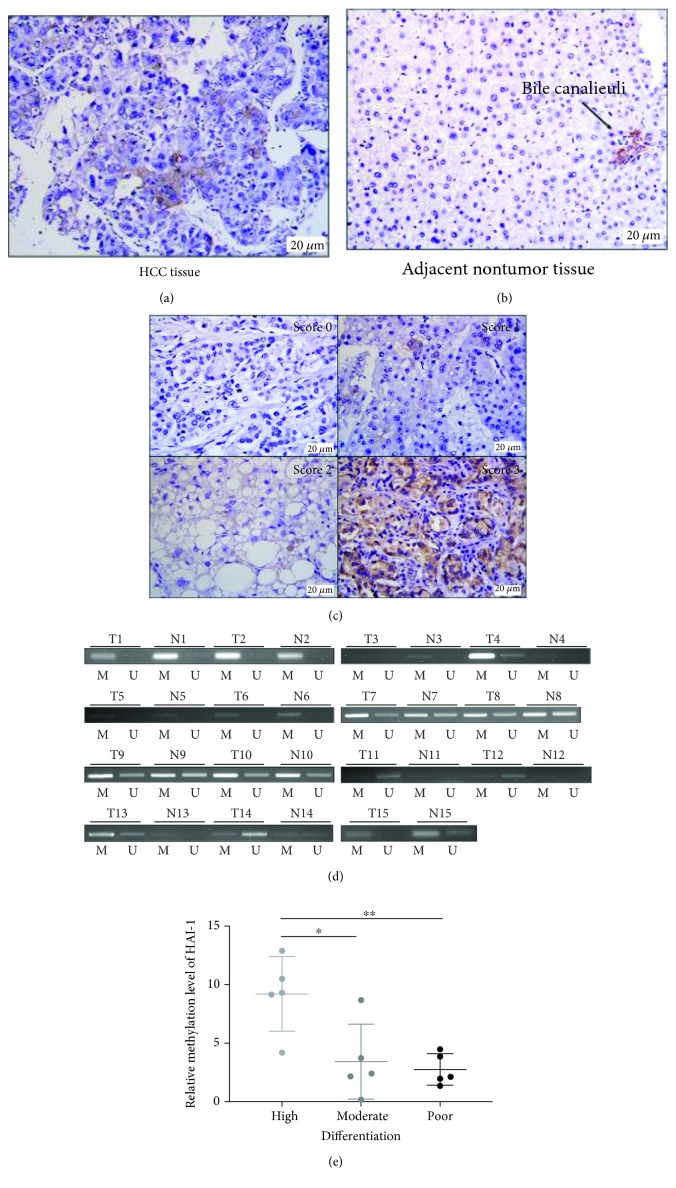
Analysis of the expression level and promoter methylation status of HAI-1 in primary HCC tissues. (a, b) Immunohistochemical staining of HAI-1 in HCC. HAI-1 was expressed in the cell membrane and cytoplasm. (a) Representative images of microscopic immunohistochemical staining for HAI-1 in HCC. Magnification ×400; scale bar = 20 *μ*m. (b) Representative photomicrographs of immunohistochemical staining of HAI-1 in adjacent nontumor surrounding HCC liver tissue were negative for HAI-1 expression. An arrow indicated as bile canaliculi. The biliary epithelial cells were positive for HAI-1. (c) Representative photomicrographs of HCC tissues and normal liver tissues illustrating expression levels of HAI-1 immunohistochemical staining (0, 1, 2, and 3). (d) Representative analysis of HAI-1 methylation in primary tumors (T) and paired nontumor tissues (N) by MSP. *n* = 3. (e) Relative methylation level of HAI-1 by qMSP in highly, moderately, and poorly differentiated HCC tumors. Mean ± SD; ^∗^*p* < 0.05, ^∗∗^*p* < 0.01; *n* = 3.

To confirm our hypothesis in HCC patients, an immunohistochemical stain of a pair of 15 HCC tissues and matched normal liver tissues was examined. Representative staining was shown in Figures 4(a) and 4(b). Two of HCC tissues exhibited a strong (score 3, 14T) to moderate (score 2, 12T) expression level of HAI-1, compared to cases which lost HAI-1 (score 0) in nontumor tissues (Figure 4(c)). We next designed methylation-specific PCR (MSP) primers within the 55 CpG sites of BGS to analyze HAI-1 methylation status in these 15 primary HCC tissues and paired nontumor tissues. In 26.7% (4/15) of cases, HAI-1-unmethylated bands were stronger in tumor tissues than paired normal tissues (Figure 4(d)). Samples 12 and 14 with a high expression level of HAI-1 amplified a strong unmethylated band which indicated that promoter hypomethylation is responsible for upregulated HAI-1 expression in HCC tissues. Furthermore, we performed qMSP to show the correlation between methylation and expression level of HAI-1 in HCC tissues and summarized in Supplementary Materials ([Supplementary-material supplementary-material-1]). It showed that the HCC tissues with expressed HAI-1 present a relatively low methylation level of HAI-1. qMSP results also showed that methylation levels of HAI-1 in highly differentiated tissues were significantly higher than that in the moderately or poorly differentiated tissues (*p* < 0.05 and *p* < 0.01, respectively) (Figure 4(e)).

We also analyzed the association between promoter methylation status and clinicopathological characteristics, illustrated in Table 2. The methylation status of the HAI-1 promoter in HCC did not show a significant association with clinicopathological parameters other than the differentiation status.

## 4. Discussion

In the present study, we confirmed that HAI-1 was expressed in the more malignant HCC cell line Hep3B but at a lower expression level or absent in less malignant HCC cell lines SMMC7721 and HepG2 and immortal normal liver cell line L02. We further demonstrated that hypomethylation status in the HAI-1 promoter region was involved in the regulation of HAI-1 expression levels in HCC cell lines and tissues. Of note, promoter hypomethylation status of HAI-1 was associated with poor tumor differentiation in HCC patients. To the best of our knowledge, this was the first attempt to investigate the regulatory mechanism of HAI-1 expression in HCC, and this was also the first time to report that the aberrant expression of HAI-1 is modulated by altered epigenetic modification via DNA hypomethylation in the cancer.

HAI-1 may play roles differently according to specific tumor microenvironment. Downregulation of HAI-1 was found in a series of cancer types, such as colorectal carcinoma, breast cancer, gastric cancer, and prostate cancer [24, 37, 38], and was associated with worse malignancy and poor prognosis. In contrast, HAI-1 expression was observed in HCC tissues but not in normal liver tissues, and its expression correlates with vascular invasion, advanced tumor stage, and worse prognosis [27, 36]. The results of this study also showed that HAI-1 was only expressed in HCC tissues.

Complicated mechanisms may be involved in HAI-1's functions under physiological and pathological conditions. HAI-1 plays important roles in placental differentiation, as HAI-1 knockout mouse showed development failure and even prenatal lethality, but it is not necessary in some mature tissues in which its expression could not be observed [15, 16]. Previous studies indicated that the expression level of HAI-1 is associated with tumor differentiation in colon cancer [39] and thyroid cancer [40]. Although we did not analyze the correlation between HAI-1 expression and HCC clinicopathological variables in this work, it is important to investigate in future work.

Previous studies have demonstrated that epigenetic modification, especially DNA methylation, was involved in hepatocarcinogenesis and survival outcomes of HCC patients [30, 41] both at a genome-wide level and tumor-related single gene [34, 42]. Herein, we showed that the aberrant methylation status in the promoter region of HAI-1 was associated with inverse HAI-1 expression in HCC cell lines (Table 1) and that HAI-1 expression due to hypermethylation was elevated after demethylation. The BGS analysis results indicated that L02 and SMMC7721 cell lines maintained almost the same methylation frequency but a higher expression level of HAI-1 in SMMC7721 than in L02 (22.1-fold, Table 1). The (q)MSP results showed that some HCC tissues with a negative HAI-1 expression have hypomethylated promoter status ([Supplementary-material supplementary-material-1]), suggesting that other epigenetic regulations may be involved. It is reported that miR-221/222 was responsible for HAI-1 expression in gastric cancer [43]. In addition, we noticed that histone deacetylation inhibitor TSA also could increase the HAI-1 expression in SMMC7721 cell line ([Supplementary-material supplementary-material-1]), suggesting that other mechanisms including the histone deacetylation modulation may lead to the induced expression of HAI-1 in HCC. Further work needs to be done to confirm the current results and check whether another regulation mechanism was involved in hepatocellular carcinoma, using more STR profiling-validated cell lines.

Here, we presented HAI-1 promoter hypomethylation in a proportion of HCC patients and linked expression upregulation. Built upon the several published whole-genome bisulfite analysis studies which described that methylation alterations play a role in inhibiting differentiation in hematopoietic malignancies [44, 45], we did observe that this epigenetic change in HAI-1 contributes reduced differentiation ability in this retrospective HCC case cohort. While data in the current study provided some evidence, the association between the promoter methylation level of HAI-1 and HCC differentiation status needs to be further confirmed in a larger patient cohort.

In conclusion, we confirmed an elevated HAI-1 expression in some hepatocellular carcinomas compared to normal liver tissues. Our observation of hypomethylation of HAI-1 promoter in HCC cell lines and patient sample provides an explanation for abnormal HAI-1 expression, and this epigenetic aberration is correlated with worse tumor differentiation. This study supports future explorations of HAI-1 biology in HCC with a better understanding of the regulation mechanism.

## Figures and Tables

**Table 1:** Methylation frequency of HAI-1 CpG sites and expression level of HAI-1 in HCC cell lines.

Cell lines	Relative expression level of HAI-1	HAI-1 methylation status in sequenced CpGs	
		Unmethylated (%)	Methylated (%)
L02	1.08 ± 0.47	3.6	96.4
SMMC7721	23.89 ± 4.72	2.4	97.6
Hep3B	2597 ± 377	79.0	21.0

**Table 2:** Frequency of HAI-1 CpG island hypomethylation in hepatocellular carcinomas and their association with clinical variables.

Variables		HAI-1 methylation status		*p* value
		Hypomethylation (*n* = 7)	Hypermethylation (*n* = 8)	
Gender	Male	7	6	0.155
	Female	0	2	

Age (years)	<58	4	6	0.464
	≥58	3	2	

Tumor size (cm)	<5	1	4	0.143
	≥5	6	4	

Tumor number	Single	7	6	0.155
	Multiple	0	2	

Liver cirrhosis	No	4	1	0.067
	Yes	3	7	

HBV infection	No	3	2	0.464
	Yes	4	6	

Vascular invasion	No	5	7	0.438
	Yes	2	1	

Differentiation	High	0	5	*0.031*
	Moderate^1^	4	1	*0.010*
	Poor^2^	3	2	*0.038*

TNM stage	I+II+IIIA/B	6	7	0.919
	IIIC+IV	1	1	

^1^High differentiation vs. moderate differentiation. ^2^High differentiation vs. poor differentiation.

## Data Availability

The data used to support the findings of this study are available from the corresponding authors upon request.

## Supplementary Materials

Supplementary Materials Figure S1: relative methylation level and expression level of HAI-1 in HCC tissues. The horizontal axis indicated each HCC tissue. The forward vertical axis indicated the relative methylation level of HAI-1 by qMSP, and the backward vertical axis indicated the expression score of HAI-1 by IHC. Figure S2: histone deacetylation inhibitor TSA increased the HAI-1 expression level in the SMMC7721 cell line in a dose-dependent manner.
