# Supplementary material for: Promoter Hypomethylation Is Responsible for Upregulated Expression of HAI-1 in Hepatocellular Carcinoma
Source: Dis Markers. 2019 Aug 28;2019:9175215. doi: 10.1155/2019/9175215 (PMC6735181; doi:10.1155/2019/9175215)
Supplement: Supplementary Materials — Figure S1: relative methylation level and expression level of HAI-1 in HCC tissues. The horizontal axis indicated each HCC tissue. The forward vertical axis indicated the relative methylation level of HAI-1 by qMSP, and the backward vertical axis indicated the expression score of HAI-1 by IHC. Figure S2: histone deacetylation inhibitor TSA increased the HAI-1 expression level in the SMMC7721 cell line in a dose-dependent manner. [file 9175215.f1.pdf]

Disease Markers

Promoter hypomethylation is responsible for upregulated expression of HAI-1 in hepatocellular carcinoma

Xiaoxiao Du, [1, 2] Lingyan Wu,[1] Muhammad S. U. Rahman,[1] Xiaodong Teng,[3] Lisong Teng,[2] Jingjia Ye,[4] and Jiang Cao[1]

Supplementary Materials

Figure S1: Relative methylation level and expression level of HAI-1 in HCC tissues.

Figure S2: Histone deacetylation inhibitor TSA increased HAI-1 expression level in SMMC7721 cell line in a dose dependent manner.

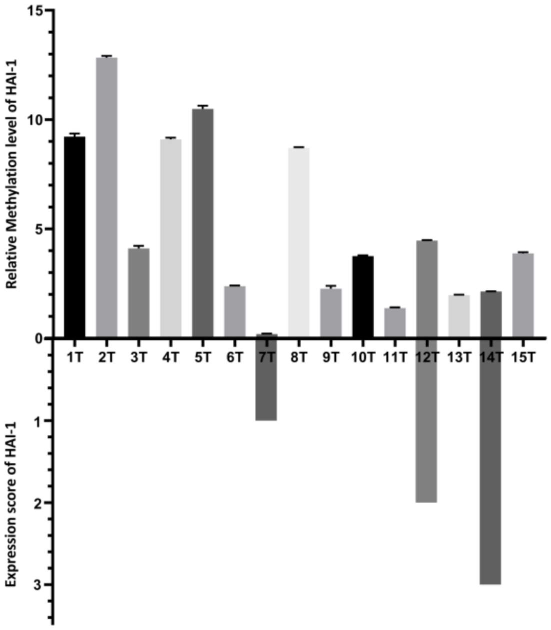

Figure S1: Relative methylation level and expression level of HAI-1 in HCC tissues. The horizontal axis indicated each HCC tissue. The forward vertical axis indicated the relative methylation level of HAI-1 by qMSP and the backword vertical axis indicated the expression score of HAI-1 by IHC.

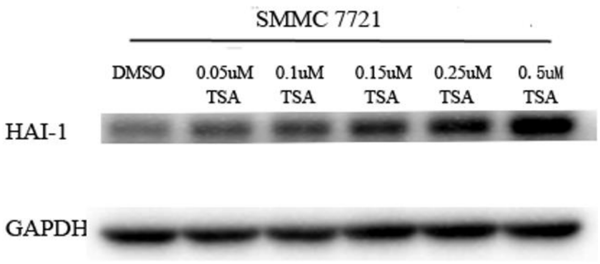

Figure S2: Histone deacetylation inhibitor TSA increased HAI-1 expression level in SMMC7721 cell line in a dose dependent manner.
